# Galangin, a Flavonoid from Lesser Galangal, Induced Apoptosis via p53-Dependent Pathway in Ovarian Cancer Cells

**DOI:** 10.3390/molecules25071579

**Published:** 2020-03-30

**Authors:** Haizhi Huang, Allen Y. Chen, Xingqian Ye, Rongfa Guan, Gary O. Rankin, Yi Charlie Chen

**Affiliations:** 1 College of Life Sciences, China Jiliang University, Hangzhou 310018, China; huangtian205@163.com; 2 College of Science, Technology & Mathematics, Alderson Broaddus University, Philippi, WV 26416, USA; 3 Department of Pharmacy Informatics, Seattle Children’s Hospital, Seattle, WA 98101, USA; al50chen@gmail.com; 4 College of Biosystems Engineering and Food Science, National-Local Joint Engineering Laboratory of Intelligent Food Technology and Equipment, Zhejiang University, Hangzhou 310058, China; psu@zju.edu.cn; 5 College of Food Science and Engineering, Zhejiang University of Technology, Hangzhou 310014, China; rongfaguan@163.com; 6 Department of Biomedical Sciences, Joan C. Edwards School of Medicine, Marshall University, Huntington, WV 25755, USA; rankin@marshall.edu

**Keywords:** apoptosis, galangin, antioxidants, ovarian cancer, western blot, flavonoid

## Abstract

Among women worldwide, ovarian cancer is one of the most dangerous cancers. Patients undergoing platinum-based chemotherapy might get adverse side effects and develop resistance to drugs. In recent years, natural compounds have aroused growing attention in cancer treatment. Galangin inhibited the growth of two cell lines, A2780/CP70 and OVCAR-3, more strongly than the growth of a normal ovarian cell line, IOSE 364. The IC_50_ values of galangin on proliferation of A2780/CP70, OVCAR-3 and IOSE 364 cells were 42.3, 34.5, and 131.3 μM, respectively. Flow cytometry analysis indicated that galangin preferentially induced apoptosis in both ovarian cancer cells with respect to normal ovarian cells. Galangin treatment increased the level of cleaved caspase-3 and -7 via the p53-dependent intrinsic apoptotic pathway by up-regulating Bax protein and via the p53-dependent extrinsic apoptotic pathway by up-regulating DR5 protein. By down-regulating the level of p53 with 20 μM pifithrin-α (PFT-α), the apoptotic rates of OVCAR-3 cells induced by galangin treatment (40 μM) were significantly decreased from 18.2% to 10.2%, indicating that p53 is a key regulatory protein in galangin-induced apoptosis in ovarian cancer cells. Although galangin up-regulated the expression of p21, it had little effect on the cell cycle of the two ovarian cancer cell lines. Furthermore, the levels of phosphorylated Akt and phosphorylated p70S6K were decreased through galangin treatment, suggesting that the Akt/p70S6K pathways might be involved in the apoptosis. Our results suggested that galangin is selective against cancer cells and can be used for the treatment of platinum-resistant ovarian cancers in humans.

## 1. Introduction

Ovarian cancer is one of the most lethal gynecological cancers in the reproductive system of females, afflicting nearly 204,000 women per year and causing approximately 125,000 deaths [1]. Due to a lack of effective biomarkers for screening, most patients with ovarian cancer are diagnosed at late stages. The first-line therapy might cure 80% of patients with ovarian cancer, but the cancer will recur in 70% of those patients with aggressive or advanced tumors, with a limited hope for survival [2]. Though platinum drugs have been most commonly used for treating ovarian cancer, patients often have adverse side effects when receiving platinum drug therapy. More selective drugs are required to overcome high rates of normal cell toxicity and resistance of cancer cells to conventional platinum-based chemotherapy. In recent years, considerable attention has been directed toward the effectiveness of natural products in cancer treatment [3,4].

**Figure 1:**
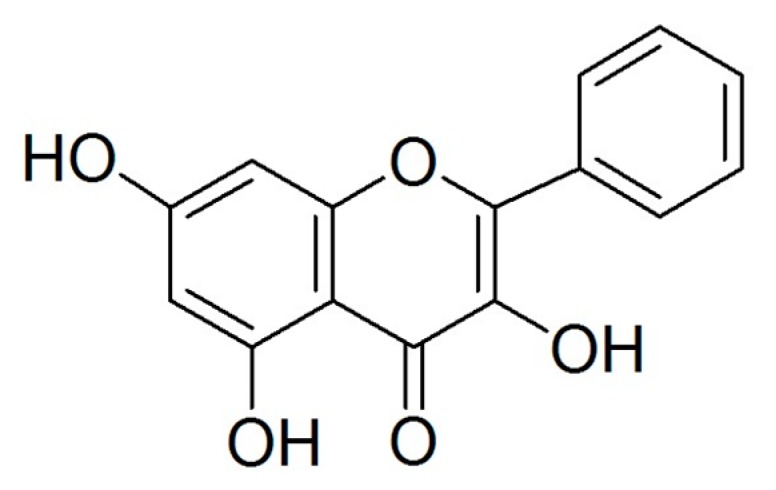
Structure of galangin.

Flavonoids, a type of natural polyphenol, can be found in various foods, especially in vegetables and fruits. Flavonoids prevent oxidation and inflammation, induce apoptosis, and diminish angiogenesis and cell proliferation showing high anticancer activity [5,6,7,8]. Galangin (Figure 1), which can be found in the galangal rhizome and propolis, is recognized as an antioxidant polyphenol, a member of the flavonol subclass of flavonoids. Previous studies have reported that galangin induces apoptosis in melanoma cells as well as in hepatocellular and gastric carcinoma cells [9,10,11]. Our previous study suggested that galangin inhibits ovarian cancer cell angiogenesis [7]; however, its effect of inducing apoptosis on ovarian cancer cells needs further study.

There are two types of chemoresistance by mechanism, i.e., oncogenic resistance and non-oncogenic resistance. Oncogenic resistance, in which some downstream apoptotic pathways are blocked, is also considered as apoptosis avoidance resistance [12]. Apoptosis, recognized as a process of programmed cell death, occurs in multicellular organisms, being critical for cell homeostasis. Cancer therapy resistance can be overcome by inducing apoptosis in ovarian cancer cells [13]. Many mechanisms are connected to inducing apoptosis in ovarian cancer cells, which include the promotion of caspase enzymatic activities, changes of the mitochondrial permeability pore, up-regulation of the pro-apoptotic protein Bax, down-regulation of the anti-apoptotic protein Bcl-2 and enhanced expression of p53 protein. According to the previous work, p53 is critical for the mechanism of apoptosis [14], activating a wide network of signals, including the extrinsic pathways of apoptosis (e.g., Killer/DR5) and the intrinsic pathways of apoptosis (e.g., Bcl-2 protein family, Noxa and Puma) [15]. Thus, the up-regulation of the expression of p53 might be an effective method for the treatment of tumors through inducing apoptosis.

As published in a previous paper [7], galangin had a cytotoxic effect on two platinum-resistant ovarian cancer cell lines, i.e., A2780/CP70 and OVCAR-3, in vitro. But the mechanisms are still unknown. In this study, we examined the rates of apoptosis and cell cycle arrest in the two ovarian cancer cells which were treated with galangin. Finally, the pathways involved in the effects of galangin were investigated. 

## 2. Results

### 2.1. Galangin Inhibits Proliferation of Ovarian Cancer Cells

**Figure 2:**
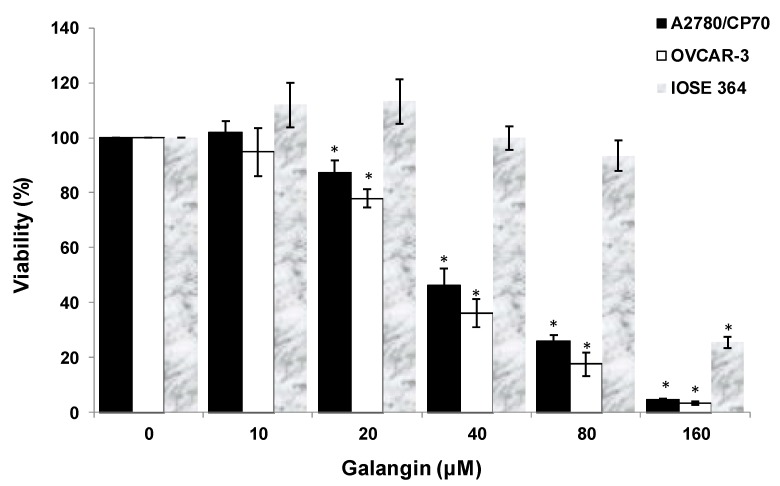
Effect of galangin on the viability of ovarian cells. Cell viability was determined by 3-(4,5-dimethylthiazol-2-yl)-2,5-diphe-nyltetrazolium bromide (MTT)-based method and expressed as percentages of the control. All cells (1 × 10^4^/well) were seeded into 96-well plates and incubated overnight before being treated with galangin at different concentrations. * *p* < 0.05 as compared to the control.

After treatment of ovarian cells with galangin, the cytotoxic effects of galangin on the platinum-resistant ovarian cancer cells were investigated by 3-(4,5-dimethylthiazol-2-yl)-2,5-diphe-nyltetrazolium bromide (MTT) assay. The treatment of ovarian cancer cells A2780/CP70 and OVCAR-3 with galangin resulted in a concentration-dependent decrease in the cell viability over 24 h. The MTT assay suggested that the cell viability with the galangin treatment (10–160 μM) for 24 h ranged from 102.0% ± 4.1% to 4.7% ± 0.3% (*p* < 0.05) for A2780/CP70 cells (Figure 2). Likewise, OVCAR-3 cell viability was also reduced with various concentrations. Cell viability decreased from 94.8% ± 8.7% at a concentration of 10 μM galangin to 3.4% ± 0.5% at a concentration of 160 μM galangin (*p* < 0.05) (Figure 2). The growth-inhibitory activity of galangin on normal ovarian cells IOSE 364 was also detected (Figure 2). The IC_50_ values of galangin on A2780/CP70, OVCAR-3 and IOSE 364 cells were estimated as 42.3, 34.5, and 131.3 μM, respectively. It was observed that galangin had a lower growth-inhibitory activity in IOSE 364 cells than in A2780/CP70 and OVCAR-3 cells, suggesting that galangin is more cytotoxic to platinum-resistant ovarian cancer cells than to normal ovarian cells.

### 2.2. Galangin Inhibits Ovarian Cancer Cells Proliferation in Chicken Chorioallantoic Membrane (CAM)

**Figure 3:**
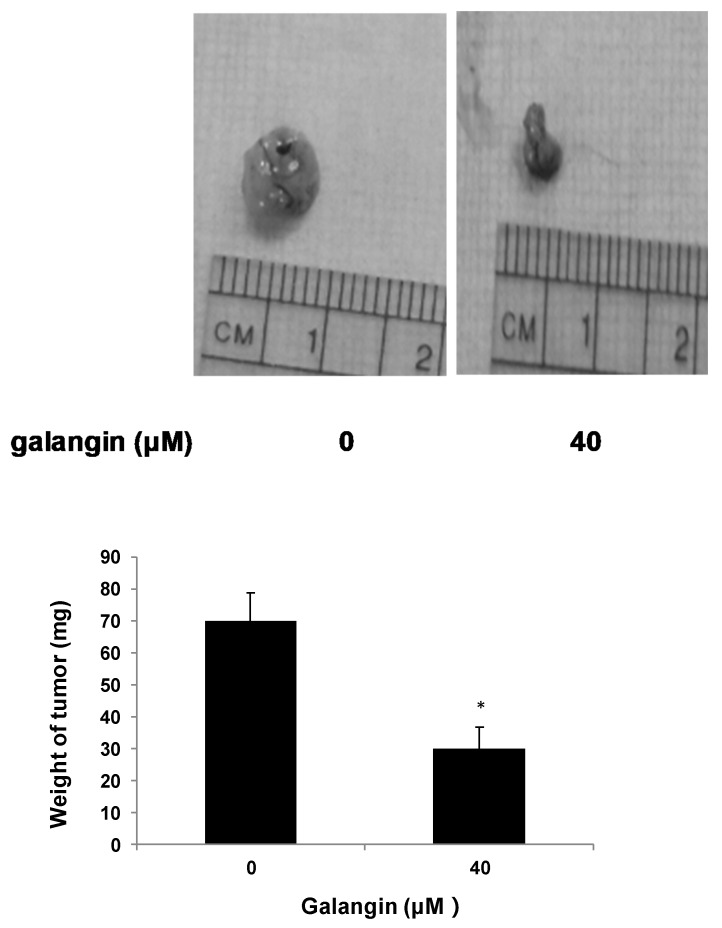
Effect of galangin on the proliferation of ovarian cancer cells by CAM assay. Aliquots of OVCAR-3 cells (20 μL, 6 × 10^7^/mL) were mixed with 80 μL of Matrigel and supplemented with 0- or 40-µM galangin. The mixture was implanted onto the CAM of the 9-day-old chicken embryo. Chicken embryos were incubated for another 5 days. Tumor implants were weighed using an electronic scale. * *p* < 0.05 as compared to the control.

The CAM assay was performed to determine the effect of galangin on ovarian cancer cell proliferation in vivo. Figure 3 shows that the typical OVCAR-3 cell tumors were lighter and smaller when treated with 40 μM galangin when compared with corresponding controls, suggesting that galangin inhibits OVCAR-3 cell growth in CAM.

### 2.3. Effects of Galangin on Apoptosis and Cell Cycle in A2780/CP70 and OVCAR-3 Cells

**Figure 4:**
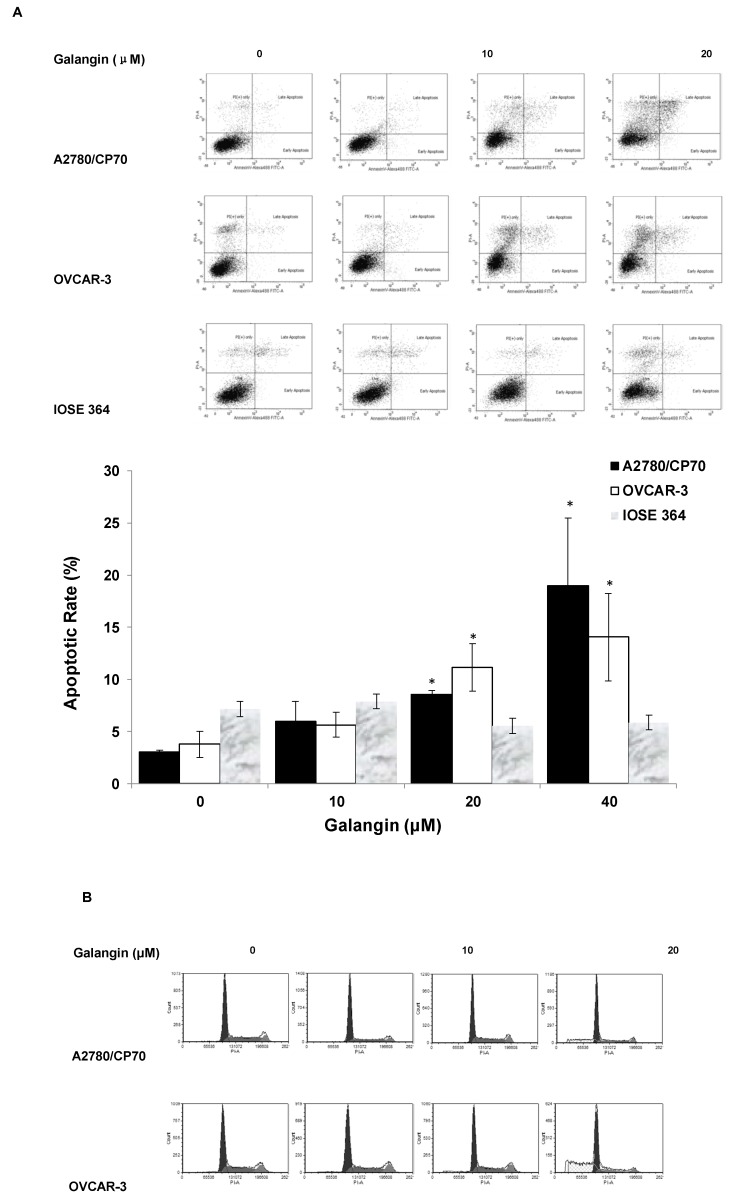
Effect of galangin on apoptosis and the cell cycle in ovarian cancer cells A2780/CP70 and OVCAR-3. (**A**) A2780/CP70, OVCAR-3 and IOSE 364 cells were treated with galangin at different concentrations for 24 h, and the apoptotic rates were measured by flow cytometry. * *p* < 0.05 as compared to the control. (**B**) A2780/CP70 and OVCAR-3 treated with galangin at different concentrations for 24 h are analyzed by flow cytometry after PI staining.

To judge whether apoptosis induction was responsible for the inhibitory effects of galangin on ovarian cancer cells, we investigated the apoptosis rates of a normal ovarian cell line, IOSE 364, and ovarian cancer cell lines, A2780/CP70 and OVCAR-3, treated with galangin (10–40 μM) for 24 h. The cells were stained with Annexin V and Propidium Iodide (PI), and then analyzed by flow cytometry. Figure 4A shows that galangin significantly led to apoptosis of ovarian cancer cells A2780/CP70 and OVCAR-3 in a concentration-dependent manner, whereas it had little effect on the apoptosis induction in normal ovarian cells, IOSE 364.

The cell cycle phase distribution of cells treated with various concentration of galangin (10–40 μM) was analyzed by flow cytometry after PI staining. Figure 4B shows that galangin had little impact on the cell cycle in A2780/CP70 and OVCAR-3 cells, suggesting that galangin may inhibit ovarian cancer cells through a mechanism separate from cell cycle arrest.

### 2.4. The Pathways of Galangin-Induced Apoptosis in Ovarian Cancer Cells

**Figure 5:**
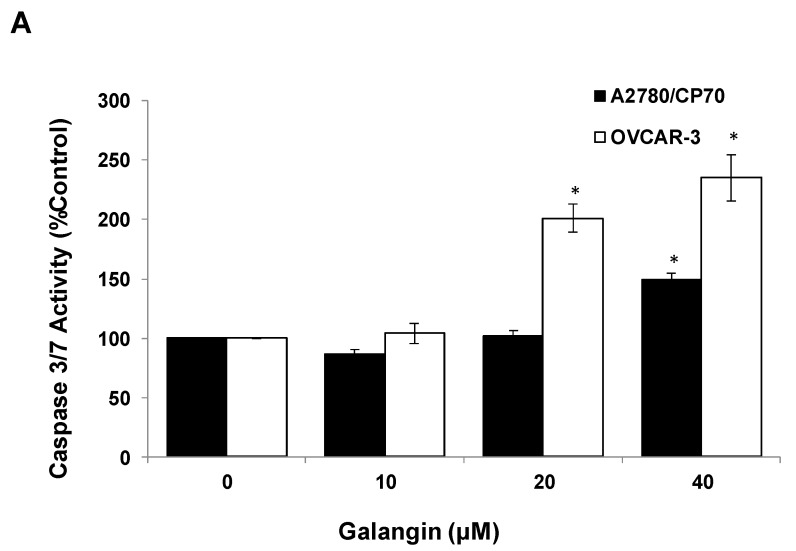

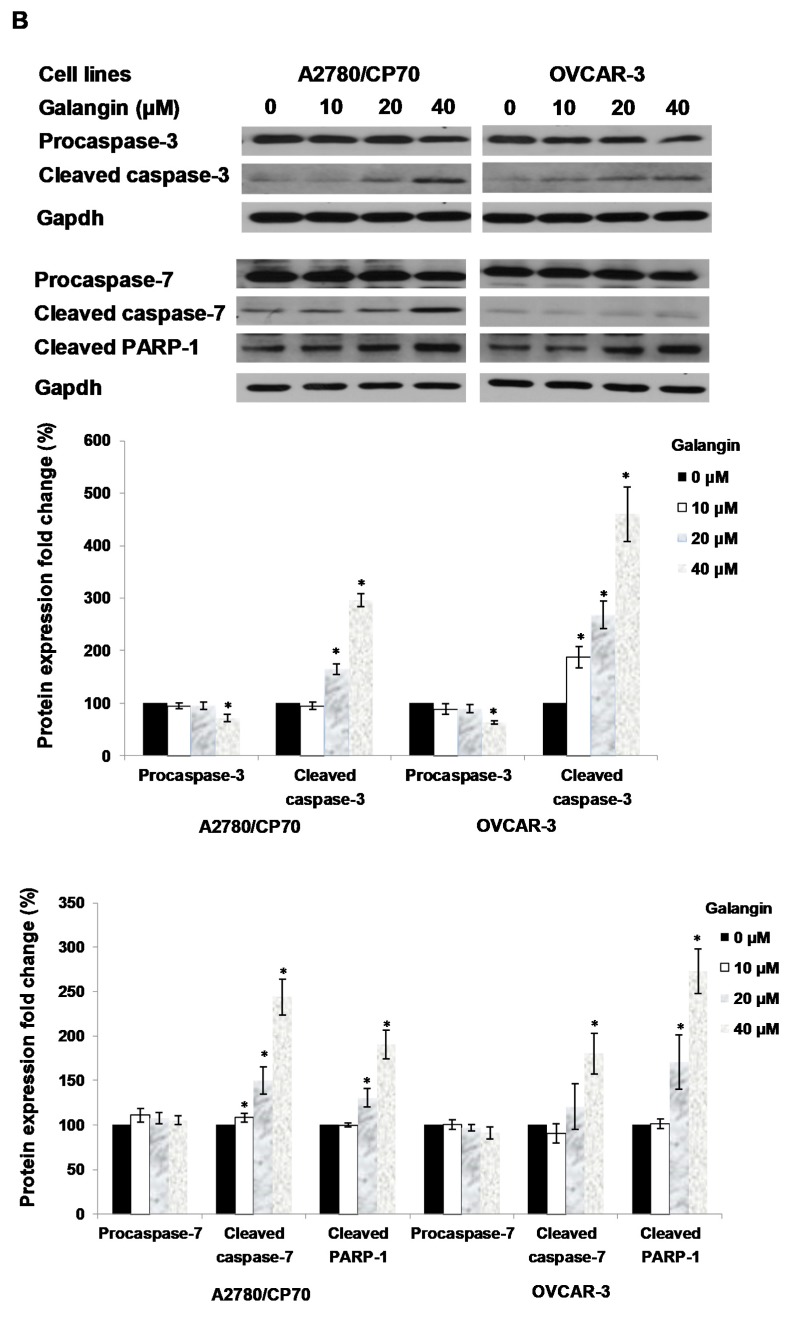

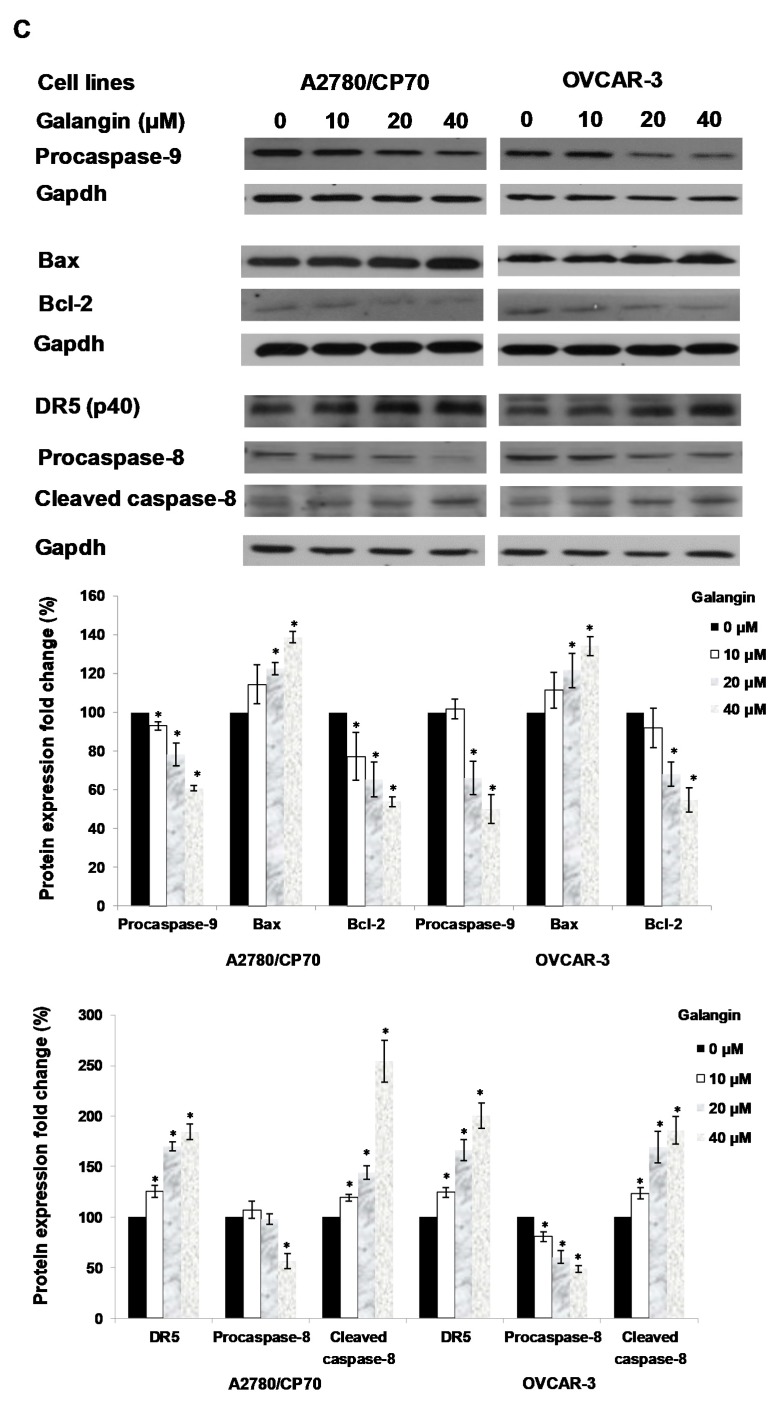
Pathways of galangin-induced apoptosis in A2780/CP70 and OVCAR-3 cells. (**A**) After being treated with galangin for 8 h, the caspase-3/7 enzymatic activities in A2780/CP70 and OVCAR-3 cells were investigated using a Caspase-Glo 3/7 Assay kit. (**B**) Galangin down-regulated the levels of procaspase-3, -7, and up-regulated the levels of cleaved caspse-3, -7 and PARP-1 in A2780/CP70 and OVCAR-3 cells. (**C**) Galangin down-regulated the levels of procaspase-8, -9 and Bcl-2, and up-regulated the levels of cleaved caspse-8, matured DR5 (p40) and Bax in A2780/CP70 and OVCAR-3 cells. Protein bands were quantitated, normalized to corresponding Gapdh bands and expressed as percentages of control. * *p* < 0.05 as compared to the control.

The Caspase-Glo 3/7 Assay kit and Western blot assay were employed after A2780/CP70 and OVCAR-3 cells were treated with galangin. Figure 5A shows that when cells were treated with 40 μM galangin for 8 h, caspase-3/7 enzymatic activities were maximally increased to 1.48 ± 0.06-fold and 2.35 ± 0.20-fold in A2780/CP70 and OVCAR-3 cells, respectively, as compared with controls. Figure 5B shows that galangin significantly up-regulated the levels of cleaved caspase-3, caspase-7 and PARP-1, and down-regulated the levels of procaspase-3 and procaspase-7 in the two ovarian cancer cell lines.

Next, proteins which were involved in galangin-induced apoptosis in A2780/CP70 and OVCAR-3 cells were examined. Figure 5C shows that the Bcl-2 and procaspase-9 protein levels were down-regulated, and that Bax protein expression was up-regulated when treated with galangin. After the activation from proprotein, the cleaved caspase-9 might be degraded so that it was not detected. Galangin up-regulated the expression of matured DR5 and cleaved caspase-8 and down-regulated the levels of procaspase-8 (Figure 5C). The above results suggested that caspase, Bax, Bcl-2, DR5-dependent pathways might be involved in galangin-induced apoptosis in A2780/CP70 and OVCAR-3 cells.

### 2.5. The Key Protein p53 in the Regulation of Galangin-Induced Apoptosis.

**Figure 6:**
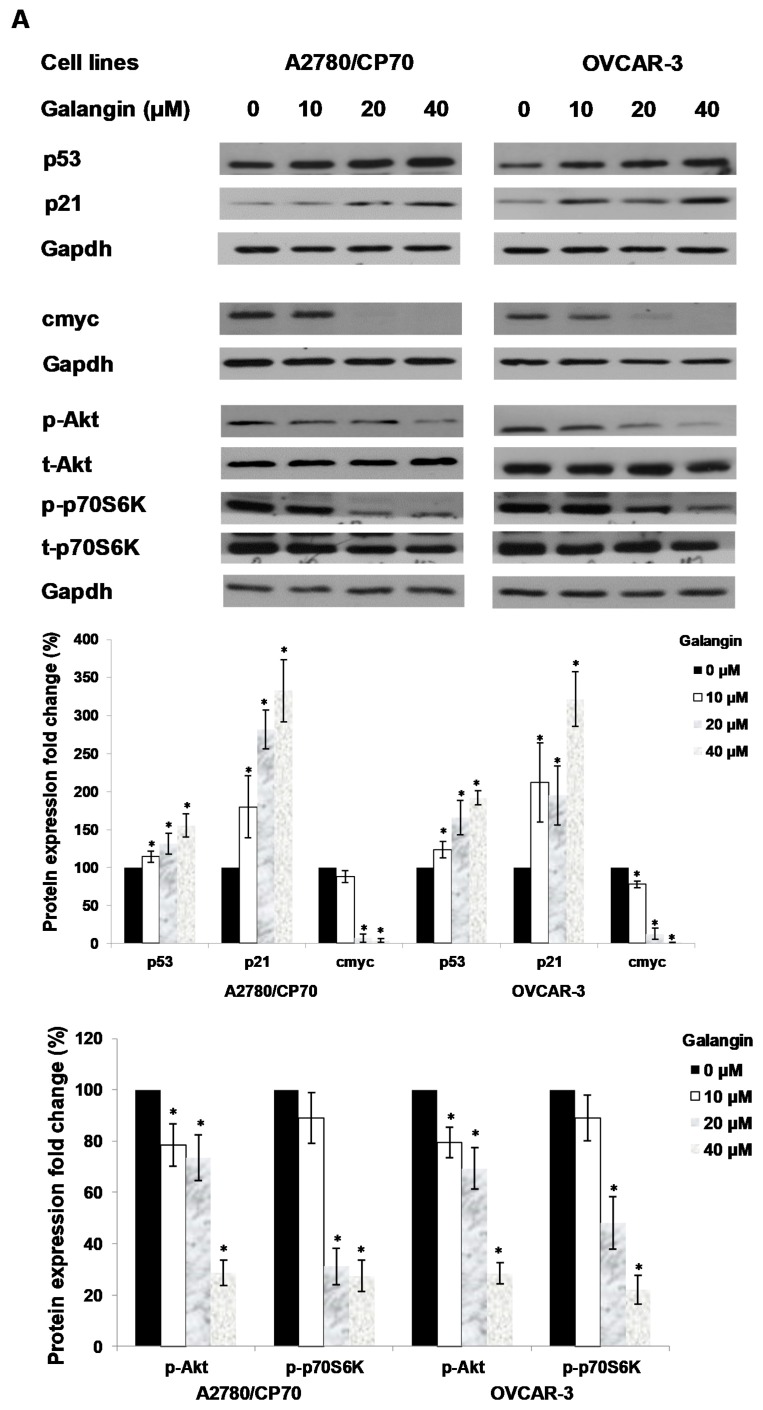

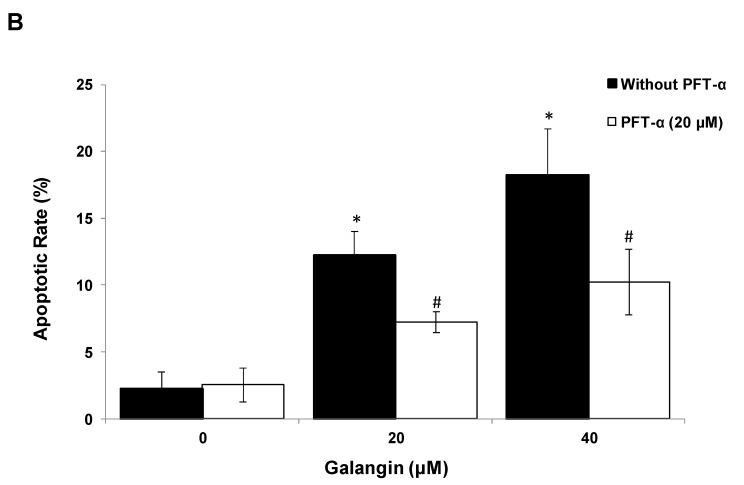

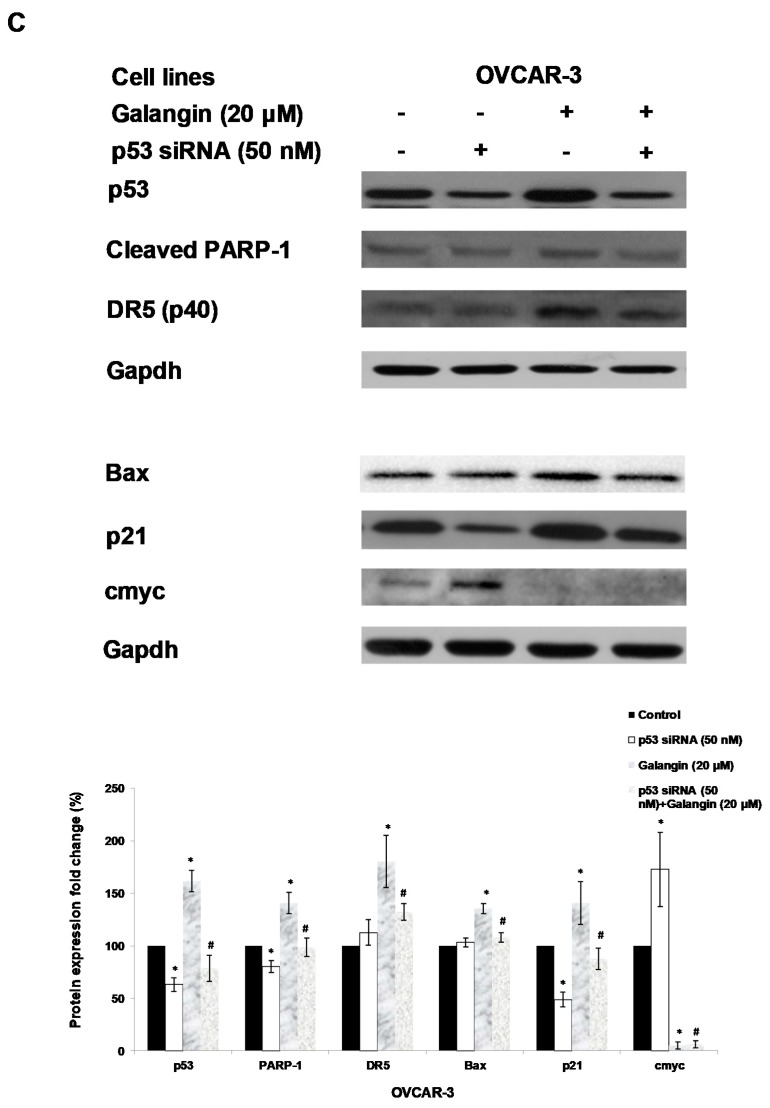
The key protein p53 in the regulation of the galangin-induced apoptosis in A2780/CP70 and OVCAR-3 cells. (**A**) Galangin up-regulated p53 and p21 protein expressions, and down-regulated cmyc, p-Akt and p-p70S6K protein levels. (**B**) OVCAR-3 cells were treated with p53 inhibitor PFT-α and galangin for 24 h. The apoptotic rates were measured using a fluorescence microscope (ZEISS) after Hoechst 33,342 staining. (**C**) The galangin-increased levels of cleaved PARP-1, matured DR5, Bax and p21 proteins were abrogated after knockdown of p53 protein. Protein bands were quantitated, normalized to corresponding Gapdh bands and expressed as percentages of the control. * *p* < 0.05 as compared to the control; # *p* < 0.05 as compared to the galangin-treated control.

An experiment was performed to find out which proteins regulated galangin-induced apoptosis in A2780/CP70 and OVCAR-3 cells. Hence, we investigated the levels of p-Akt, t-Akt, p-p70S6K, t-p70S6K, p53, cmyc and p21 proteins. Figure 6A shows that galangin up-regulated p53 and p21 protein expressions and down-regulated p-Akt, p-p70S6K and cmyc protein levels.

Using p53 siRNA and PFT-α, we investigated the role of p53 on apoptosis induction in OVCAR-3 cells and found that the apoptotic rates of OVCAR-3 cells induced by galangin treatment (40 μM) were significantly decreased from 18.2% ± 3.5% to 10.2% ± 2.5% when treated with 20 μM PFT-α (Figure 6B). Figure 6C shows that the knockdown of p53 (p53 siRNA) caused the attenuation of galangin-increased levels of cleaved PARP-1, matured DR5, Bax and p21 proteins. The knockdown of p53 up-regulated the levels of cmyc protein in OVCAR-3 cells. The cmyc protein was not detected when OVCAR-3 cells, pre-cultured with p53 siRNA or control siRNA, were treated with galangin. These results suggested that p53 may play an important role in the regulation of galangin-induced apoptosis in OVCAR-3 ovarian cancer cells.

## 3. Discussion

Among women worldwide, ovarian cancer is one of the most fatal cancers. Platinum-based chemotherapy is one of the broadly used methods for the treatment of ovarian cancer. However, some side effects and resistance to drugs have seriously limited the treatment of ovarian cancer. In recent years, there has been an increased interest in natural products as ovarian cancer treatments. Flavonoids are widespread in many types of plants or plant products and have been reported to have anticancer properties in ovarian cancer cells [16,17]. Though galangin shows anticancer activity in several cancer cell lines (e.g., hepatocellular, gastric and colon carcinoma cells) [9,10,18], the effect of galangin on ovarian cancer remains unclear. In this study and previous published data [7], we found that galangin effectively inhibits ovarian cancer cell lines, OVCAR-3 and A2780/CP70, whereas it has less of a cytotoxic effect on a normal ovarian cell line, IOSE 364. Compared with our previous work [19], galangin is more cytotoxic to the two platinum-resistant ovarian cancer cells than cisplatin. Previous studies reported that flavonoids could hardly reach low micromolar levels in plasma and organs due to their low bioavailability [20]. For future studies, some strategies, such as nanoemulsion and nanoliposome [21], should be used to increase the bioavailability of galangin.

Studies on the cancer cell inhibition have often emphasized apoptotic mechanisms because of their significance for the activities of cells and the maintenance of regular cellular functions. Resistance to apoptosis might be a possible reason why cancer cells develop resistance to chemotherapy drugs [12]. It was reported that agents in pre-clinical disease models, targeting the apoptotic pathway, make tumor cells sensitive to cancer chemotherapy and radiotherapy [22]. The results of the flow cytometry assay suggested that galangin induces apoptosis in ovarian cancer cells A2780/CP70 and OVCAR-3 over normal ovarian cells IOSE 364. However, we found that galangin only induced apoptosis in approximately 20% of cancer cells even at the concentration of 40 mM and hardly affected the cell cycle; the reason for a more than 50% reduction in formazan absorbance by 40 mM galangin is unclear. Other mechanisms, such as the induction of senescence or an increase in death due to necrosis, may be associated with the cytotoxicity of galangin to ovarian cancer cells. Both caspase-3 and caspase-7, belonging to the cysteine-aspartic acid protease (caspase) family, serve as critical executioner proteins that mediate the cleavage of PARP, thereby regulating apoptosis in cells. In this study, galangin treatment up-regulates cleaved caspase-3, cleaved caspase-7 and cleaved PARP-1 to induce apoptosis in both A2780/CP70 and OVCAR-3 cell lines. Our results are consistent with a previous study that showed that galangin stimulates apoptosis in glioblastoma multiforme cells and up-regulates the levels of cleaved PARP-1 and cleaved caspase-3,7 [23]. However, in their study, the IC_50_ values of galangin on the viability of glioblastoma multiforme cells were above 200 μM, higher than our results in ovarian cancer cells.

Some activation mechanisms strictly regulate the initiation of apoptosis [24]. There are two major pathways associated with apoptosis, i.e., the intrinsic (the mitochondrial) apoptotic and the extrinsic (the death receptor) apoptotic pathways. The intrinsic apoptosis regulator Bax is a member of the Bcl-2 family, being critical for the mediation of apoptosis by binding to and antagonizing the Bcl-2 protein. It reacts with pore proteins, thereby increasing the permeability of the membrane to activate caspase-9, an initiator caspase related to the intrinsic pathway [25,26]. The extrinsic apoptotic pathway is regulated through the tumor necrosis factor-related apoptosis-inducing ligand (TRAIL) engaging its respective death receptor, e.g., DR5. Caspase-8 and -10, initiator caspases having been linked to the extrinsic death pathway, are activated by DR5. Both caspase-3 and -7 are activated after the activation of either the intrinsic or extrinsic pathway. In this study, we showed that galangin might induce apoptosis through the Bcl-2/Bax-associated intrinsic pathway in A2780/CP70 and OVCAR-3 cells. Our results are consistent with previous work reporting that galangin up-regulates the expression of Bax protein and induces apoptosis in hepatocellular carcinoma cells [10]. We also found that galangin activates caspase-8, the DR5-associated extrinsic pathway of apoptosis. It was reported that changes in the balance of Bcl-2/Bax that directly control the permeability of mitochondria are involved in the differential induction of apoptosis in cancer versus normal cells [27]. Furthermore, normal cells are resistant to TRAIL. Thus, targeting DR5 may be a good strategy that can selectively eliminate tumor cells [28]. The selective induction of apoptosis in both ovarian cancer cell lines in this study might result from its effects on the expression of Bcl-2, Bax and DR5 proteins.

The multifunctional tumor suppressor p53 is associated with several steps of cancer cell carcinogenesis, e.g., DNA repair, transcription, differentiation, cell cycle arrest, senescence, angiogenesis and apoptosis [29]. Once the p53 gene is damaged, tumor suppression will be under serious threat. It has been reported that more than 50% of p53 genes in human tumors are mutated or deleted [30]. In ovarian cancer cells, p53 also has been reported to play an important role in the mechanism of apoptosis [14]. Therefore, it may be a good strategy for the treatment of ovarian tumors or prevention of metastasis to increase the amount of p53 protein. This increase could promote apoptosis through a wide network of signals including extrinsic pathways of apoptosis, e.g., Killer/DR5, and intrinsic pathways of apoptosis, e.g., Bax, Puma and Noxa [14,31]. In some other cancer cells, the extrinsic pathway of apoptosis associated with DR5 is also regulated by p53 [32]. Our results suggested that the galangin-activated apoptosis in ovarian cancer cells, which is regulated by p53, might be related to the DR5-associated extrinsic pathway. Additionally, previous work suggested that the loss of the p53 function is always associated with relatively low levels of Bax, a transcriptional target of the p53 gene. The Bcl-2/Bax genes are regulated by p53 in many types of cancer cells, including ovarian cancer cells [33]. Our data suggested that p53 regulates Bax-associated apoptosis induced by galangin. These results suggested that the protein p53 is critical for both the galangin-activated intrinsic and extrinsic pathways of apoptosis. A previous work reported that galangin induces apoptosis in human nasopharyngeal carcinoma cells in a p53-independent pathway [34]. It was not consistent with our results, which reported that galangin regulates p53 to induce apoptosis in ovarian cancer cells. The different mechanisms of apoptosis may result from the different cancer cell lines.

Many cancer cells have been reported to exhibit defective cell-cycle checkpoints, leading to uncontrolled proliferation and growth [35]. It is clear that p21, tightly regulated by the tumor suppressor p53 protein, controls cell cycle progression at the G1 and S phases [36]. p21 may play a pro-apoptotic role in either a p53-dependent or independent manner, and it has been suggested that p21 is a negative regulator of p53-dependent apoptosis. In this study, galangin up-regulates the levels of p21 and p53 in A2780/cp70 and OVCAR-3 ovarian cancer cells, but has little effect on the cell cycle. p21 effects are mediated by p53, and it seems to have an association with galangin-induced apoptosis but not cell cycle arrest in ovarian cancer cells. Akt can be activated by phosphorylation and mediates the p70S6K proteins [37], leading to the regulation of the cancer cells’ survival by inhibiting apoptosis and cell cycle arrest [38,39]. The results of this study showed that galangin reduces the levels of phosphorylated Akt and phosphorylated p70S6K in the two cancer cell lines, suggesting that the induction of apoptosis may also be involved in the Akt/p70S6K pathways.

## 4. Materials and Methods

### 4.1. Cell Culture and Treatment

IOSE 364, normal ovarian surface epithelial cells extracted from healthy women and immortalized with SV40 T/t, were provided by Dr. N. Auersperg, University of British Columbia, VA, Canada. A2780/CP70 and OVCAR-3, human ovarian cancer cell lines, were provided by Dr. B. Jiang, Department of Microbiology, Immunology, and Cell Biology, West Virginia University, Morgantown, WV, USA. Culture medium for the three ovarian cell lines included RPMI 1640 medium (Sigma-Aldrich, St. Louis, MO, USA) supplemented with 10% US-qualified fetal bovine serum (Invitrogen, Grand Island, NY, USA). Cells were kept in a humidified incubator (Thermo Fisher Scientific, Waltham, MA, USA) at 37 °C with 5% CO_2_. Galangin was dissolved in dimethyl sulfoxide (DMSO) at a concentration of 100 mM as stock solutions, and equal amounts of DMSO were controlled for each experiment.

### 4.2. Reagents and Antibodies

Galangin was purchased from Shanghai Yuanye Bio-Technology (Shanghai, China). The primary antibodies against Bcl-2, p-Akt, t-Akt, p-p70S6K, t-p70S6K, Bax, p21, p53, DR5, caspase-3, -7, -8 and -9 were purchased from Cell Signaling Technology, Inc. (Dancers, MA, USA). The primary antibodies against Gapdh, cmyc and p53 were purchased from Santa Cruz Biotechnology Inc. (Santa Cruz, CA, USA).

### 4.3. Cell Viability

By determining the 3-(4,5-dimethylthiazol-2-yl)-2,5-diphe-nyltetrazolium bromide (MTT) dye absorbance, we investigated the effect of galangin on cell proliferation. Human ovarian cancer cells, A2780/CP70 and OVCAR-3, and normal ovarian cells, IOSE 364, (1 × 10^4^/well) were seeded into 96-well plates with 5% CO2 at 37 °C and incubated overnight before being treated with galangin at different concentrations (10–160 μM) in quintuplicates for another 24 h. Cells were treated with an equal volume of DMSO only as the control group. The cells were washed with PBS after the medium was removed. Subsequently, 100 μL MTT (1 mg/mL) was added to each well and incubated with 5% CO2 at 37 °C in the dark for 4 h. Formed formazan crystals were dissolved in 200 μL DMSO before the supernatant was removed, and the OD values were measured at 570 nm. Cell viability was expressed as the percentage of the control group (DMSO only) of five separate experiments.

### 4.4. Flow Cytometry Analysis of Cell Cycle

A2780/CP70 and OVCAR-3 cells (1 × 10^6^/dish) were treated with galangin at different concentrations (10–40 μM) for 24 h, digested by trypsin and collected through centrifugation. Subsequently, cells were washed with cold PBS, suspended in 70% ethanol and stored at −20 °C. After being collected through centrifugation for 6 min at 1000 rpm, the cell pellets were washed with PBS again, and the PBS was removed. Then, they were incubated with 180 μg/mL RNase A at 37 °C for 15 min. Next, 50 μg/mL propidium (final concentration) was added for 15 min staining and then detection by flow cytometry (FACSCalibur system, BD Biosciences, Franklin Lakes, NJ, USA). Data were plotted and analyzed with FCS Software (De Novo Software, Los Angeles, CA, USA).

### 4.5. Flow Cytometry Analysis of Cell Apoptosis

We determined the apoptotic effects of galangin on ovarian cancer cells, A2780/CP70 and OVCAR-3, and normal ovarian cancer cells, IOSE 364, using Alexa Fluor 488 Annexin V/Dead Cell Apoptosis Kits from Invitrogen (Grand Island, NY, USA). All cells (1 × 10^6^/dish) were treated with galangin (10–40 μM) for 24 h. The mediums were collected, and cells were digested, washed with cold PBS and re-suspended in binding buffer. After an aliquot of 100 μL of the cell solution (1 × 10^5^ cells) was transferred to a 5 mL tissue culture tube, 5 μL of FITC Annexin V and 1 μL Propidium Iodide (PI) were added to the cells. Subsequently, the cells were vortexed gently and incubated at ambient temperature in the dark for 15 min. Before being analyzed by flow cytometry (FACSCalibur system, BD Biosciences), each tube of the samples had 400 μL of 1× binding buffer added.

### 4.6. Apoptosis Assessment by Hoechst 33,342 Staining

OVCAR-3 cells were seeded into 60-mm dishes at a concentration of 1 × 10^6^/dish and incubated overnight before being treated with galangin/pifithrin-α (PFT-α) (Sigma-Aldrich) at different concentrations for another 24 h. Next, cells were washed with cold PBS and stained with 10 μg/mL Hoechst 33,342 (Sigma-Aldrich) dissolved in PBS in a dark room at 37 °C for 10 min. Apoptosis in OVCAR-3 cells was determined using a fluorescence microscope (ZEISS, Oberkochen, Germany).

### 4.7. Western Blot

Human ovarian cancer cells, A2780/CP70 and OVCAR-3, were seeded into 60-mm dishes at a density of 1 × 10^6^/dish and incubated overnight before being treated with galangin at different concentrations (10–40 μM) or DMSO for 24 h. After being washed with PBS, cells were lysed in extraction reagent (100 µL Mammalian Protein Extraction Reagent (M-PER, Pierce, Rockford, IL, USA), 1 µL Halt Protease, 1µL Phosphatase Inhibitor and 2 µL EDTA). A BCA Protein Assay Kit (Pierce) was employed to detect total protein levels. Protein bands were quantitated using NIH ImageJ software (National Institutes of Health, Bethesda, USA) before being normalized to corresponding Gapdh bands for analysis. The data were expressed as a percentage of the untreated (DMSO only) control value. 

### 4.8. Caspase-3/7 Assay

A2780/CP70 and OVCAR-3 cells were seeded into 96-well plates at a density of 1 × 10^4^/well and incubated overnight with 5% CO_2,_ at 37 °C before being treated with galangin at different concentrations (10–40 μM) in triplicate for another 8 h. The caspase 3/7 enzymatic activities in both ovarian cancer cell lines were detected using the Caspase-Glo 3/7 Assay kit (Promega, Madison, WI, USA). Total protein levels were used to normalize enzymatic activities. The data were expressed as a percentage of the untreated (DMSO only) control. 

### 4.9. Transfection with Small Interfering RNA (siRNA)

OVCAR-3 cells (5 × 10^5^/dish) were incubated overnight and transfected with p53 siRNA or control siRNA (Santa Cruz) using jetPRIME^TM^ DNA and siRNA Transfection Reagent (VWR International, Radnor, PA, USA) for 24 h following the manufacturer’s protocol. After the transfection, cells were treated with galangin (20 μM) or DMSO for another 24 h, and subsequently collected with extraction reagent, i.e., 100 µL Mammalian Protein Extraction Reagent (M-PER, Pierce), 1 µL Halt Protease, 1 µL Phosphatase Inhibitor and 2 µL EDTA, for a Western blot to test for Bax, cmyc, p21, DR5 and PARP-1 proteins, respectively.

### 4.10. Chicken Chorioallantoic Membrane (CAM) Assay

Specific pathogen-free fertile chicken eggs (Charles River Laboratories, North Franklin, CT, USA) were kept at 37.5 °C in an incubator and slowly turned by an automatic egg turner (G.Q.F. Manufacturing Company, Savannah, GA, USA). The OVCAR-3 cells were allowed to grow to 70% confluence and were harvested, washed with PBS and then re-suspended in serum-free medium. Aliquots of the cells (20 μL, 6 × 10^7^/mL) were mixed with 80 μL of Matrigel (BD Biosciences, San Jose, CA, USA) and supplemented with 0- or 40-µM galangin. The mixture was implanted onto the CAM of the 9-day-old chicken embryo after being pre-gelled on an autoclaved silicone mat for 30–40 min. Chicken embryos were incubated for another 5 days. We weighed the tumor implants using an electronic scale.

### 4.11. Statistical Analysis

Experiments were performed independently at least three times. The results were analyzed with Microsoft Excel (2007) (Microsoft, Redmond, WA, USA) and expressed as the mean ± standard error of mean (SEM). To examine the specific differences between each treatment and control, the results were analyzed by conducting a one-way analysis of variance (ANOVA) and by performing a post hoc test (2-sided Dunnett’s test) using SPSS (Version 18.0 for Windows) (IBM SPSS Software, Armonk, NY, USA). A p value of less than 0.05 was considered significant.

## 5. Conclusions

In conclusion, our study suggested that galangin has selective cell growth inhibition effects on two cisplatin-resistant ovarian cancer cell lines with respect to normal cells through the induction of apoptosis. For these two ovarian cancer cell lines, galangin induced p53-dependent apoptosis, which might be related to the Bcl-2/Bax-associated intrinsic pathway and the DR5-associated extrinsic pathway. However, galangin had little impact on the cell cycle in these two ovarian cancer cells. Moreover, the levels of phosphorylated Akt and phosphorylated p70S6K were reduced through galangin treatment, suggesting that the apoptosis in the two ovarian cancer cells might be involved in the Akt/p70S6K pathways. On the whole, galangin appears to be a potential candidate for the chemoprevention of ovarian cancer in humans. Because of the low bioavailability of flavonoids, future studies on the effect of galangin and the increase of bioavailability using animal models are needed to further determine the efficiency of this natural compound as an agent for the chemoprevention of ovarian cancer.
